# Lactic Acid from CO_2_: A Carbon Capture and Utilization Strategy Based on a Biocatalytic Approach

**DOI:** 10.1021/acs.est.3c05455

**Published:** 2023-12-11

**Authors:** Albert Carceller, Marina Guillén, Gregorio Álvaro

**Affiliations:** Department of Chemical, Biological and Environmental Engineering, Universitat Autònoma de Barcelona, Bellaterra, Catalonia 08193, Spain

**Keywords:** multienzymatic systems, carbon capture and utilization, biocatalysis, carbon dioxide

## Abstract

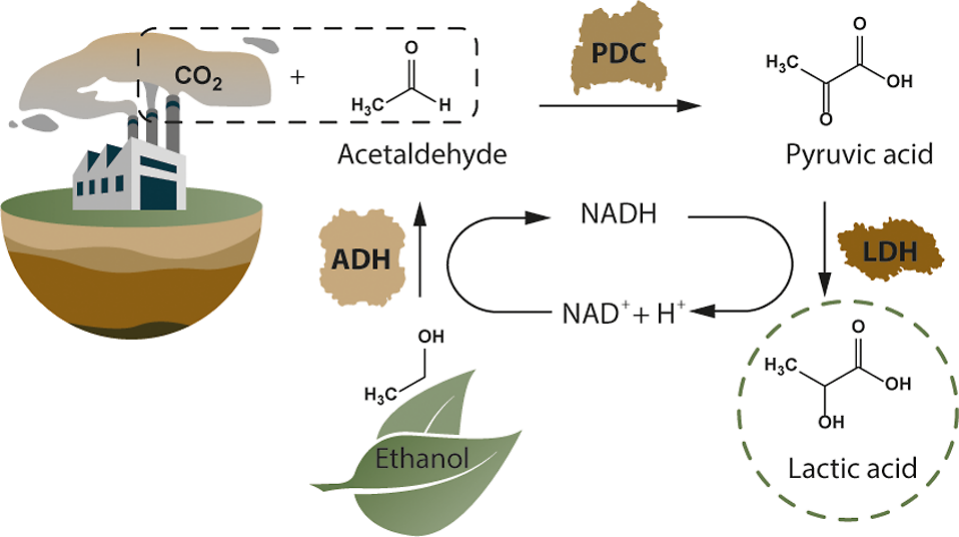

The EU low-carbon economy aims to reduce the level of CO_2_ emission in the EU to 80% by 2050. High efforts are required to achieve this goal, where successful CCU (Carbon Capture and Utilization) technologies will have a high impact. Biocatalysts offer a greener alternative to chemical catalysts for the development of CCU strategies since biocatalysis conforms 10 of the 12 principles of green chemistry. In this study, a multienzymatic system, based on alcohol dehydrogenase (ADH), pyruvate decarboxylase (PDC), and lactate dehydrogenase (LDH), that converts CO_2_ and ethanol into lactic acid leading to a 100% atom economy was studied. The system allows cofactor regeneration, thus reducing the process cost. Through reaction media engineering and enzyme ratio study, the performance of the system was able to produce up to 250 μM of lactic acid under the best conditions using 100% CO_2_, corresponding to the highest concentration of lactic acid obtained up to date using this multienzymatic approach. For the first time, the feasibility of the system to be applied under a real industrial environment has been tested using synthetic gas mimicking real blast furnace off-gases composition from the iron and steel industry. Under these conditions, the system was also capable of producing lactic acid, reaching 62 μM.

## Abstract 2

The use of CO_2_ as a feedstock for the synthesis of chemicals can contribute to carbon recycling, with a consequent reduction of fossil fuel needs while reducing greenhouse gas emissions. This study reports a biocatalytic CCU strategy to produce lactic acid.

## 1 Introduction

The Intergovernmental Panel on Climate Change (IPCC) presented a special report on the impacts of global warming considering that it has to be kept below 1.5 °C above preindustrial levels to efficiently response to the threat of climate change.^1^ The EU low-carbon economy roadmap states that EU should cut emissions to 80% below 1990 levels by the middle of the century.^2^ Therefore, according to the EU guidelines, significant efforts should be made to reduce CO_2_ emissions.

Carbon capture, utilization, and storage (CCUS) technologies involve the capture of carbon dioxide from fuel combustion or industrial processes, the transport of this carbon dioxide, and either its use as a resource to create valuable products or services or its permanent storage underground in geological formations.^3^ According to the International Energy Agency, CCUS will need to form a key pillar of efforts to put the world on the path to net-zero emissions.^4^ CCU strategies are based on the use of CO_2_ as a carbon feedstock to produce several compounds such as fuels, chemicals, and materials, obtaining a double benefit toward the climate change fight: a reduction in CO_2_ emissions and a depletion of fossil fuels as feedstock.

CO_2_ is a highly stable molecule in which carbon is in the highest valence state (+4). Besides, as it is known, the dissociation energy required to break the C=O bond in CO_2_ molecules is high (749 kJ mol^–1^), which is a thermodynamically costly reaction.^5^ Thus, high conditions of temperature or pressure and/or highly efficient catalysts are required to carry out the bond breakage.^6^

Biocatalysts represent a greener alternative to CCUs based on chemical catalysts, given that (i) they are biodegradable, safe, and nontoxic, (ii) they are produced from renewable resources, (iii) they work under mild conditions, thus leading to less energy intensive processes, (iv) they show high substrate specificity and product selectivity, and (v) there is no need of functional groups activation, protection, and deprotection steps leading to more step-economical processes and less waste generation.^7−10^ Therefore, biocatalysis is playing an important role in the development of CCUs. Several systems based on the use of enzymes to transform CO_2_ have been described in the literature: formate dehydrogenase (FDH) to transform CO_2_ into formic acid, nitrogenase MoFe protein for conversion of CO_2_ into methane, and carbonic anhydrase to convert CO_2_ into bicarbonate.^11−18^ However, most of the enzymatic systems are based on a single biocatalytic step, which limits the range of products that can be enzymatically obtained from CO_2_. Multienzymatic systems can be also applied for the transformation of CO_2_, widening the number of products of interest that can be produced compared to the use of a single enzyme. Moreover, in most cases, the enzymatic conversion of CO_2_ needs cofactors such as NAD(P)H/NAD(P)^+^ which hampered the implementation of the enzymatic systems at an industrial scale due to the high cost of these cofactors. The use of multienzymatic systems allows in situ regeneration by coupling an enzymatic cofactor regeneration reaction without the need to include chemical, electrochemical, or photochemical systems.^8,10,13−15^ Therefore, in order to widen the range of commodity chemicals that can be obtained from carbon dioxide, CO_2_-fixing enzymes has to be used as a step of a multienzymatic cascade.^10^

**Figure 1:**
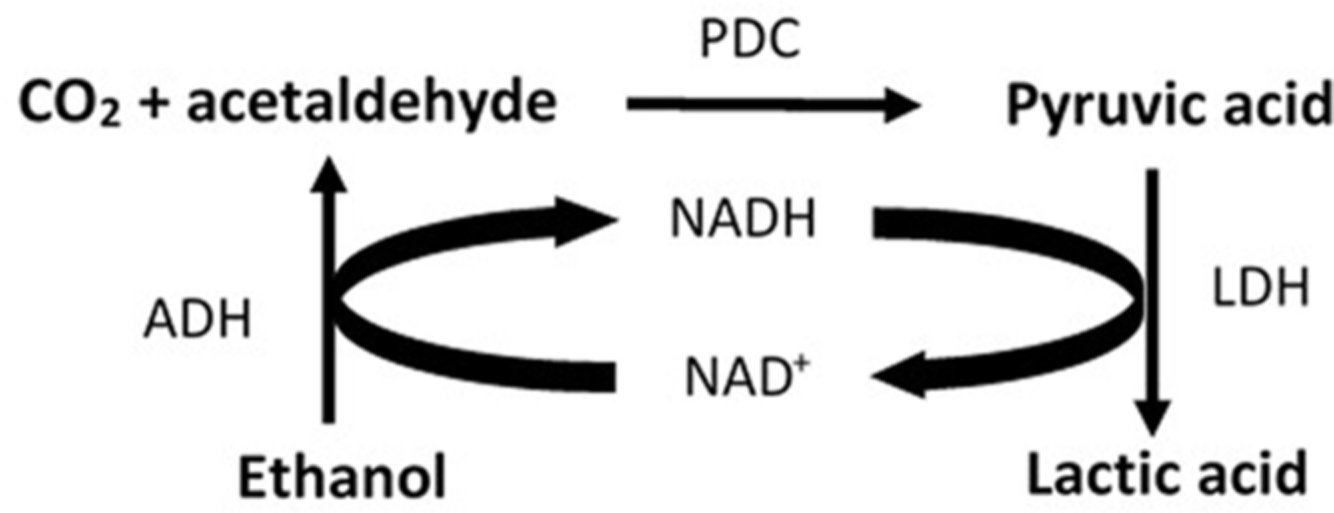
Multienzymatic system for the synthesis of lactic acid from CO_2_ and ethanol with an internal cofactor regeneration cycle. The system consists of three enzymes, ADH, PDC, and LDH.

In the present work, a multienzymatic system to produce lactic acid from CO_2_ and ethanol has been studied (Figure 1). The system is made up of three enzymes: a pyruvate decarboxylase (PDC) which converts CO_2_ and acetaldehyde into pyruvic acid, a lactate dehydrogenase (LDH) which transforms pyruvic acid into lactic acid consuming NADH, and an alcohol dehydrogenase (ADH) which produces acetaldehyde from ethanol regenerating the NADH. This third reaction allows not only cofactor regeneration but also to produce acetaldehyde from ethanol, a cheaper, less toxic, and less hazardous substrate which can also be obtained by fermentative processes from renewable resources. Following this multienzymatic system, all the reactant atoms will end up in the desired product, representing an atom economy of 100%.

In addition, the system has also been tested using synthetic gas mimicking real industrial off-gases from iron and steel industry.

## 2 Materials and Methods

### 2.1 Chemicals and Reagents

Sodium pyruvate, citric acid, sodium citrate, thiamine pyrophosphate (TPP), and magnesium chloride were obtained from Sigma Chemical Co. (St. Louis, USA) for the determination of enzyme activity and reaction media. Ethanol, potassium phosphate, sodium acetate, and sodium bicarbonate were obtained from Scharlab, S.L. (Barcelona, Spain) for the determination of enzyme activity and reaction media. Acetoin and lactic acid standards were obtained from Sigma Chemical Co. (St. Louis, USA) and used as analytical standards for gas chromatography and LC–MS analysis, respectively. NADH and NAD^+^ were obtained from Bontac Bioengineering Co. (Shenzhen, China) as reaction cofactors. Carbon dioxide gas as well as gas mixture mimicking real blast furnace off-gases composition [24.5% CO_2_, 46.6% N_2_, 23.9% CO, 1.2% O_2_, and 3.8% H_2_] was obtained from Carburos Metalicos (Barcelona, Spain). PDC from *Zymobacter palmae* (ZpPDC) was produced according to Alcover *et al*.^27^ PDC from *Saccharomyces cerevisiae* (ScPDC), ADH from Saccharomyces cerevisiae (ScADH) and Thermotoga maritima LDH (TmLDH) were produced according to Benito *et al.* (see Supporting Information).^28^

### 2.2 Enzyme Activity Assays

PDC activity was determined by coupling the PDC with ADH and following NADH oxidation at 340 nm (ε_NADH_ = 6.22 mM^–1^ cm^–1^) and 25 °C with a Varian Cary 50 Bio UV–visible spectrophotometer (Agilent). The reaction mixture contained 33 mM sodium pyruvate, 0.11 mM NADH, 3.5 U mL^–1^ of commercial ADH from *Saccharomyces cerevisiae* obtained from Sigma Chemical Co. (St. Louis, USA), 0.1 mM TPP and 0.1 mM MgCl_2_ in citrate buffer 200 mM and pH 6. One unit of enzyme activity corresponds to the amount of PDC that converts 1 μmol of pyruvate into acetaldehyde per minute. Analyses were carried out in duplicate.

LDH activity was determined by following NADH oxidation at 340 nm (ε_NADH_ = 6.22 mM^–1^ cm^–1^) and 25 °C with a Varian Cary 50 Bio UV–visible spectrophotometer (Agilent). The reaction mixture contained 33 mM sodium pyruvate and 0.11 mM NADH, in 100 mM phosphate buffer, pH 6.7. One unit of enzyme activity corresponds to the amount of LDH that converts 1 μmol of pyruvate into lactate per minute. Analyses were carried out in duplicate.

ADH activity was determined by following NAD^+^ reduction at 340 nm (ε_NADH_ = 6.22 mM^–1^ cm^–1^) and 25 °C with a Varian Cary 50 Bio UV–visible spectrophotometer (Agilent). The reaction mixture contained 567 mM ethanol and 1.7 mM NAD^+^, in phosphate buffer 100 mM at pH 8.8. One unit of enzyme activity corresponds to the amount of ADH that converts 1 μmol of ethanol into acetaldehyde per minute. Analyses were carried out in duplicate.

### 2.3 Enzyme Stability and Activity over Different pHs

Enzyme stability of ADH, PDC, and LDH was measured by incubating in 2 mL microtubes at a pH ranging from 5 to 10 for 24 h at 25 °C and 300 rpm using a Multi Therm (Benchmark Scientific Inc.) Heat Block system. For pH 5, an acetate buffer 100 mM was used, for pH 6, 7, and 8, a phosphate buffer 100 mM was used, and for pH 9 and 10, a bicarbonate buffer 100 mM was used. Samples were taken at 0, 2, and 24 h to assess enzyme activity using the corresponding activity test.

Enzyme activity at different pHs was carried out by changing the pH of the activity assay and performing the corresponding assay for each enzyme. Citrate buffer 200 mM (ZpPDC and ScPDC), 100 mM (TmLDH), or 50 mM (ScADH) for pH 6, Tris–HCl buffer 200 mM (ZpPDC and ScPDC), phosphate buffer 100 mM (TmLDH) or 50 mM (ScADH) for pH 7–8, and bicarbonate buffer 200 mM (ZpPDC and ScPDC), 100 mM (TmLDH), or 50 mM (ScADH) for pH 9–10.

### 2.4 Enzymatic Reactions

#### 2.4.1 Single-Enzyme Reactions

Each enzyme reaction was tested individually at 2 mL scale. For ADH, a bicarbonate buffer 250 mM at pH 7, 8, or 9 with ethanol 50 mM, NAD^+^ 10 mM was used. For PDC, a bicarbonate buffer 250 mM at pH 7, 8 or 9 with acetaldehyde 10 mM, TPP 1 mM, and MgCl_2_ 1 mM was used. Regarding LDH, a bicarbonate buffer 250 mM at pH 7, 8, or 9 with pyruvate 10 mM, NADH 20 mM was used. Each reaction was performed at 25 °C with 500 rpm agitation using a Multi Therm (Benchmark Scientific Inc.) heating block system for 24 h. Samples were taken after 1, 4, and 20 h (ADH reaction) or 24 h (PDC and LDH reactions).

#### 2.4.2 Lactate Dehydrogenase-Coupled Pyruvate Decarboxylase Reactions

Coupled-enzyme reactions were carried out by coupling LDH to PDC in a 50 mL reactor (Miniclave steel; Büchi) with a working volume of 25 mL. Reactions at 1 atm of CO_2_ pressure were performed in a phosphate buffer 250 mM at pH 7 with acetaldehyde 5 mM, NADH 10 mM, TPP 1 mM, MgCl_2_ 1 mM, 10 U mL^–1^ of purified PDC from *Z. palmae* (ZpPDC) or *S. cerevisiae* (ScPDC) and LDH from *T. maritima* (TmLDH) at 25 °C and 500 rpm magnetic stirring. CO_2_ was sparged into the reactor with bubbling into the reaction media. The gas outlet was opened as soon as the reaction started. Then, the outlet was closed after 5 min, when dissolved CO_2_ reached the equilibrium with the gas phase.

#### 2.4.3 Complete Multienzymatic System Reactions

Complete multienzymatic system reactions were carried out in a 50 mL reactor (Miniclave steel; Büchi) with a working volume of 20 mL. Reactions at 1 atm of CO_2_ pressure were performed in a phosphate, MOPS (3-(N-morpholino)propanesulfonic acid) , Tris-HCl or citrate buffer 250 mM at pH 7 with ethanol 1 M, NAD^+^ 10 mM, TPP 1 mM, MgCl_2_ 1 mM, using purified ADH from *S. cerevisiae* (ScADH), PDC from *S. cerevisiae* (ScPDC), and LDH from *T. maritima* (TmLDH) at 25 °C and 500 rpm magnetic stirring. CO_2_ was sparged into the reactor with bubbling into the reaction media. The gas outlet was opened as soon as the reaction started. Then, the outlet was closed after 5 min, when dissolved CO_2_ reached the equilibrium with the gas phase.

Multienzymatic test using synthetic gas mimicking blast furnace off-gases composition were carried out under optimum conditions: MOPS buffer 250 mM at pH 7, ethanol 1 M, NAD^+^ 10 mM, TPP 1 mM, MgCl_2_ 1 mM, 1 atm of synthetic gas mixture, ADH 75 U mL^–1^, PDC 150 U mL^–1^, and LDH 187.5 U/mL at 25 °C and 500 rpm agitation. The gas mixture composition was as follows according to the data provided by Arcelor Mittal in the frame of the BIOCON-CO2 project (24.5% CO_2_, 46.6% N_2_, 23.9% CO, 1.2% O_2_, and 3.8% H_2_).

### 2.5 Analytical Methods

#### 2.5.1 Acetaldehyde, Acetoin, Ethanol, and Lactic Acid Quantification

The concentration of acetaldehyde, acetoin, and ethanol in the reaction mixture were measured with a Shimadzu GC 2010 system using a Stabilwax-DA column (15 m × 0.33 × 1 μm). The conditions were injection volume, 3 μL in split mode 20:1; injector temperature, 260 °C; carrier gas, He at a constant flow of 3 mL min^–1^. The initial oven temperature, 35 °C, was held for 2 min and then programmed to increase at 15 °C min^–1^ to 120 °C, and finally programmed to increase at 40 °C min^–1^ to 240 °C, where it was held for 1 min. Before the analysis, reaction samples were inactivated by adding 20 μL of 36% (v v^–1^) HCl to 500 μL of sample.

In the case of lactic acid, the measurement of concentration was performed with a Shimadzu LCMS-2010A using an ICSep 87H USP L17 (Transgenomic) column. The conditions were as follows: buffer solution 640 μL L^–1^ of acetic acid, injection volume 5 μL, with a flow rate of 0.15 mL min^–1^; the nebulizing gas was N_2_ with a flow of 1.5 L min^–1^; the CDL temperature, 200 °C; and the heat block temperature, 200 °C. The mass-to-charge ratio was set to 89 *m*/*z* to monitor the elution of lactic acid with a running time of 25 min. Before the analysis, reaction samples were inactivated by adding 20 μL of 98% H_2_SO_4_ to 500 μL of sample.

In all analyses, compound standards of known concentration were used for the calibration of the equipment.

#### 2.5.2 Spectrophotometric NADH Analysis

NADH was measured using a Varian Cary 50 Bio UV–visible spectrophotometer (Agilent). Samples were measured in a 1.5 mL cuvette at 340 nm and 25 °C and diluted with distilled water when needed. The NADH concentration was calculated using the Lambert–Beer equation with ε_NADH_ = 6.22 mM^–1^ cm^–1^.

## 3 Results and Discussion

### 3.1 Enzyme Characterization

**Figure 2:**
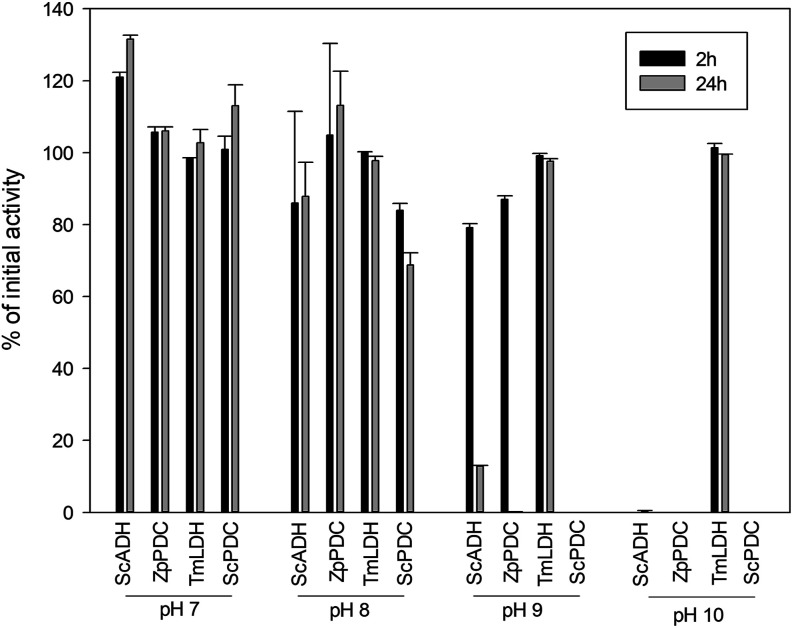
Enzyme stability at 2 and 24 h of incubation at pH 7, 8, 9, and 10 with bicarbonate buffer 100 mM at 25 °C. Alcohol dehydrogenase from *S. cerevisiae* (ScADH), PDC from *Z. palmae* (ZpPDC), PDC from *S. cerevisiae* (ScPDC), and LDH from *T. maritima* (TmLDH) were used. Error bars correspond to standard deviation (*n* = 2).

**Figure 3:**
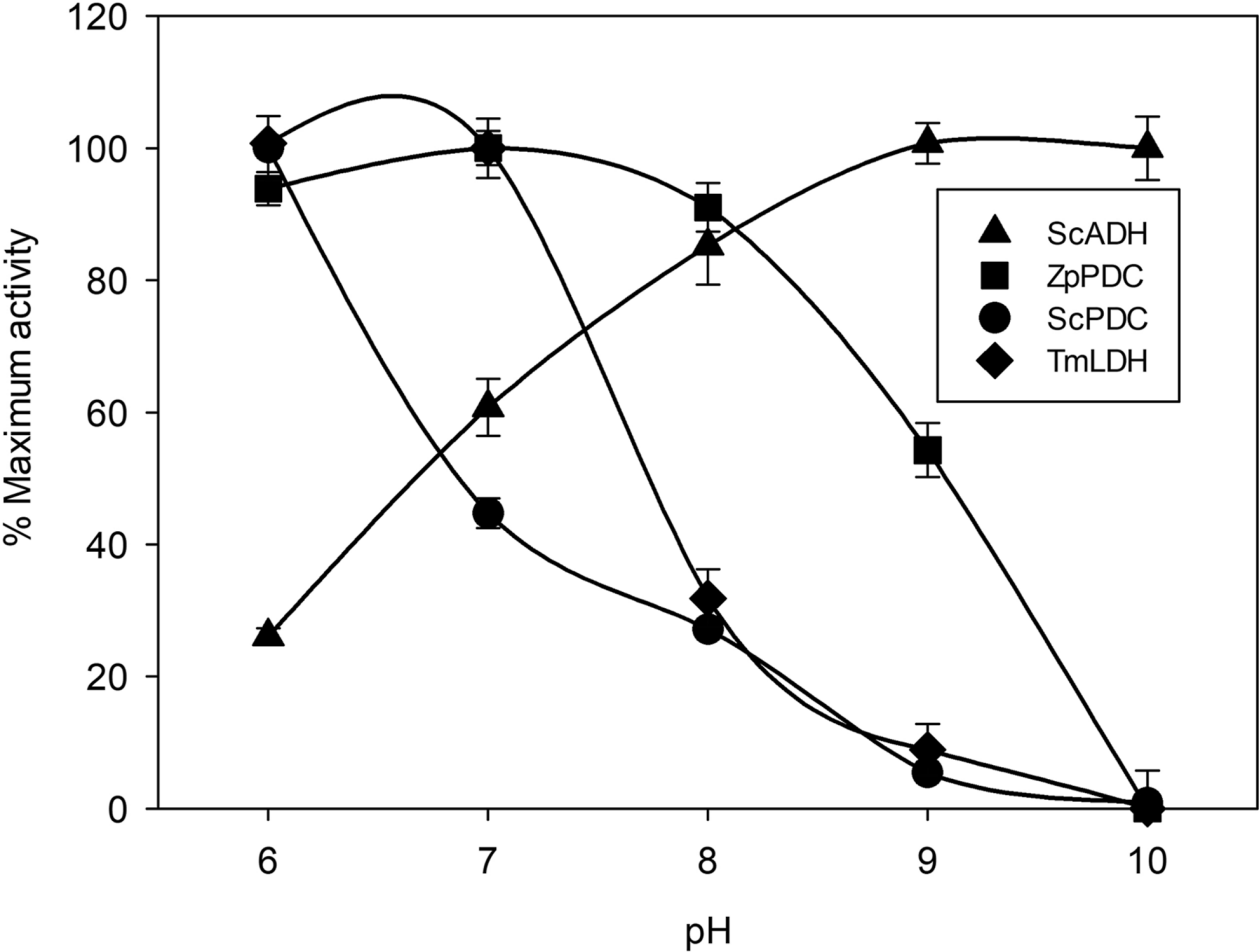
pH activity profile of ADH from *S. cerevisiae* (ScADH), PDC from *Z. palmae* (ZpPDC), PDC from *S. cerevisiae* (ScPDC) and LDH from *T. maritimae* (TmLDH) at 25 °C. Enzyme activity is expressed as a percentage of the maximum activity within the pH range. Citrate buffer 200 mM (ZpPDC and ScPDC), 100 mM (TmLDH), or 50 mM (ScADH) for pH 6, Tris–HCl buffer 200 mM (ZpPDC and ScPDC), phosphate buffer 100 mM (TmLDH) or 50 mM (ScADH) for pH 7–8, and bicarbonate buffer 200 mM (ZpPDC and ScPDC), 100 mM (TmLDH), or 50 mM (ScADH and TmLDH) for pH 9–10. Error bars correspond to standard deviation (*n* = 2).

All enzymes were characterized in terms of activity and stability toward pH aiming to determine the most suitable pH range for the study of the multienzymatic system. Aiming to work under favorable conditions for CO_2_ solubility, the pH study was performed under alkaline conditions. Regarding PDC, two enzymes were tested: a bacterial PDC from *Z. palmae* (ZpPDC) and a fungal PDC from *S. cerevisiae* (ScPDC). Both were characterized as for the ScADH and TmLDH (Figures 2 and 3).

Regarding enzyme stability, all the biocatalysts showed high activities after 24 h at pH 7–8, reaching in all cases values higher than 60% of the initial activity (Figure 2). At pH 10, all enzymes completely lost their activity after 2 h, except TmLDH which maintained 100% of its catalytic efficiency even after 24 h, probably due to its extremophile origin.^19^ At pH 9, TmLDH also showed high stability contrary to ScPDC which was completely inactive after 2 h. Even though ScADH and ZpPDC maintained up to 80% of the initial activity after 2 h, ZpPDC was inactive after 24 h and ScADH showed less than 20% of its initial activity.

The pH activity profile of all enzymes is depicted in Figure 3. ZpPDC and TmLDH showed their best performance at pH 7, while ScPDC showed its highest activity at pH 6. All three enzymes suffered an activity decrease as pH is increased up to a complete deactivation at pH 10. On the contrary, ScADH showed activities higher than 60% in all tested pHs, reaching the highest biocatalytic performance at pH 9 and 10.

According to these results, pH 10 was discarded due to inactivation of TmLDH and ZpPDC. Thus, the multienzymatic system was tested at pH 7, 8, and 9.

### 3.2 Single Enzymatic Reaction Study

Each reaction within the multienzymatic system was first tested individually at pH 7, 8, and 9, according to the results obtained in the enzyme characterization. All the tests were performed with bicarbonate buffer as the CO_2_ source.

**Figure 4:**
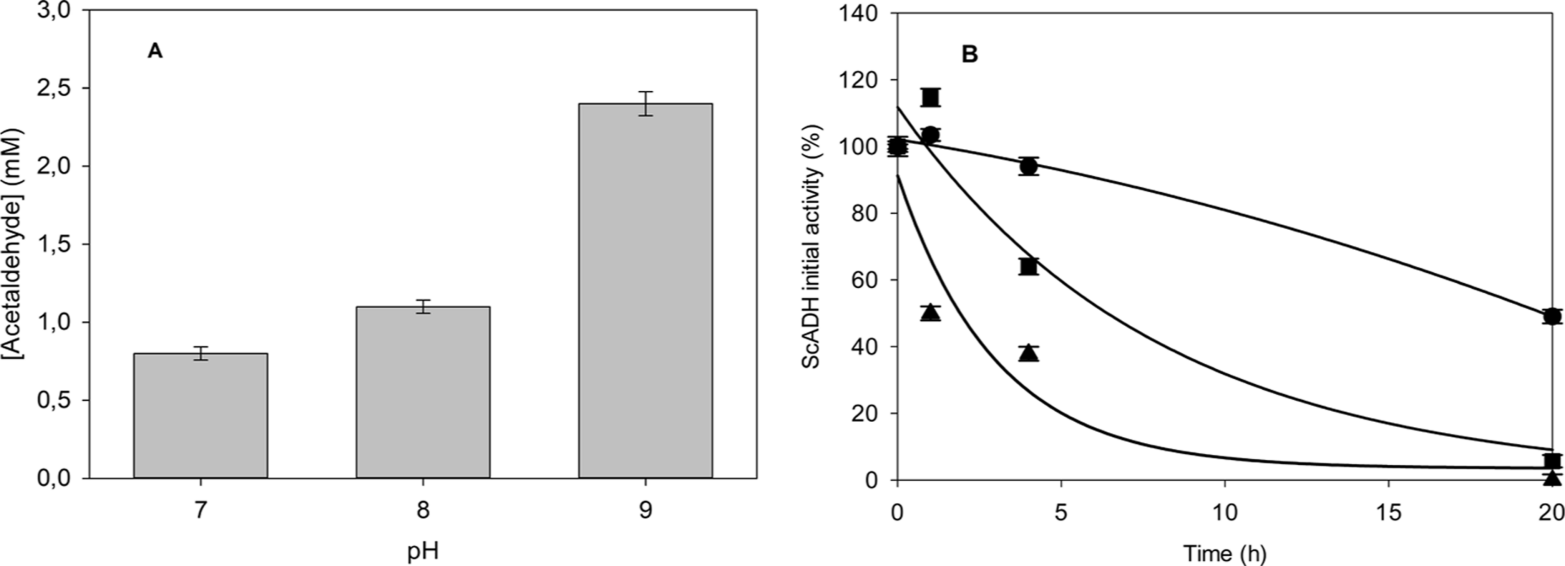
(A) Acetaldehyde formation from ethanol catalyzed by ScADH at different pHs after 20 h of reaction. (B) Stability of ADH from *S. cerevisiae* (ScADH), expressed as initial activity percentage, during the reaction at different bicarbonate buffer pHs. Reaction conditions: bicarbonate buffer 250 mM, ethanol 50 mM, NAD^+^ 10 mM, and ADH 7 U mL^–1^ at 25 °C and 500 rpm agitation. Error bars correspond to standard deviation (*n* = 2).

Figure 4A shows acetaldehyde formation from ethanol catalyzed by ADH from *S. cerevisiae* (ScADH). The highest yield was obtained at the most alkaline pH as shown in Figure 4A, reaching 2.45 mM acetaldehyde at pH 9. However, the highest enzyme stability was obtained at pH 7 with a half-life of 20 h, 20-fold higher than at pH 9 where the enzyme is completely inactivated after 20 h (Figure 4B). These results are in accordance with enzyme activity and stability profiles (Figures 2 and 3), showing that the pH has an opposite effect on the ADH activity and stability. Moreover, these results indicate a stronger effect of pH on stability than on activity (20-fold stability increase from pH 9 to 7 compared to 1.7-fold increase in activity from pH 7 to 9).

**Figure 5:**
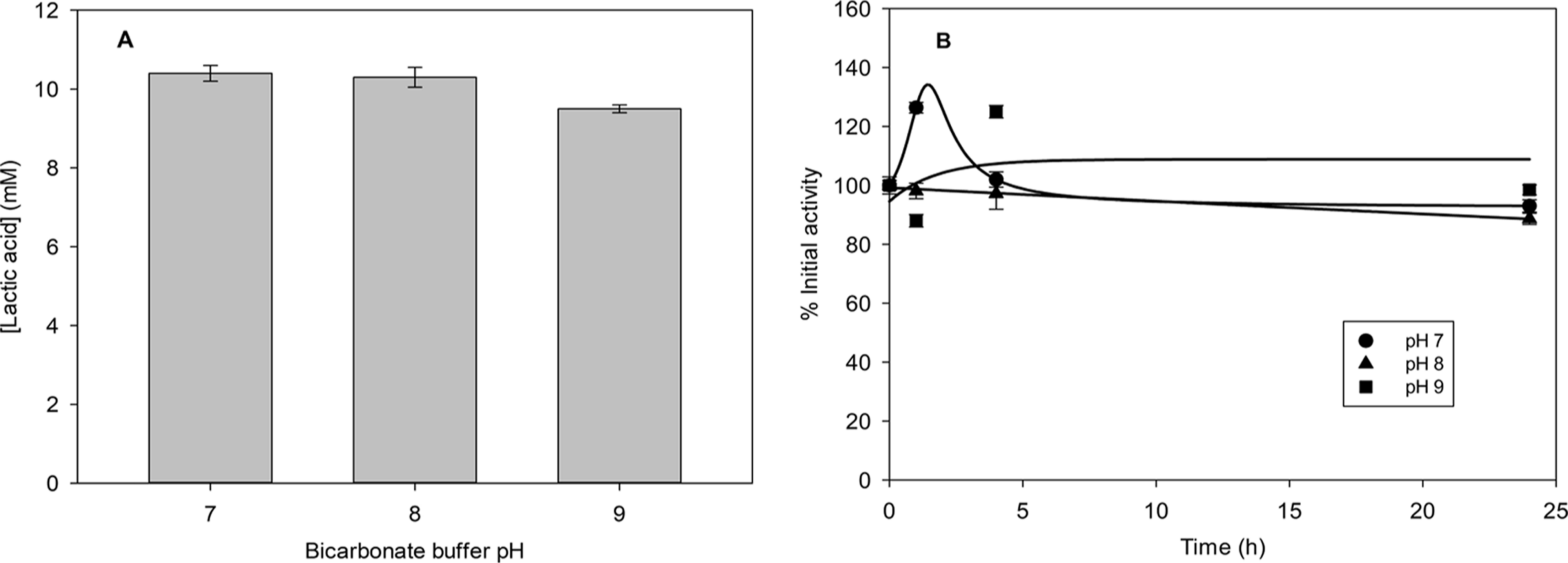
(A) Lactic acid synthesis from pyruvic acid catalyzed by TmLDH at different pHs. Concentration at different pHs after 24 h. (B) Stability of LDH from *T. maritima* (TmLDH), expressed as initial activity percentage, during the reaction at different bicarbonate buffer pHs. Reaction conditions: bicarbonate buffer 250 mM, pyruvate 10 mM, NADH 20 mM, and LDH 20 U mL^–1^ at 25 °C and 500 rpm. Error bars correspond to standard deviation (*n* = 2).

The reaction catalyzed by LDH from *T. maritima* was also studied. TmLDH catalyzed the reduction of pyruvic acid to yield lactic acid. Results are depicted in Figure 5A, showing a lower dependence of TmLDH toward pH compared to the synthesis of acetaldehyde catalyzed by ScADH (Figure 5). The highest yield was obtained at pH 7 as shown in Figure 5A, with a 100% yield and conversion. Furthermore, the LDH of *T. maritima* shows a high stability at all tested pHs (Figure 5B), suggesting that a pH with a higher activity can be chosen while keeping the enzyme stable.

The high yield obtained as well as the high stability showed at all tested pHs by TmLDH, which catalyzes the last step of the system, represents a great advantage for the development of the multienzymatic system by shifting the equilibriums toward the lactic acid formation.

**Figure 6:**
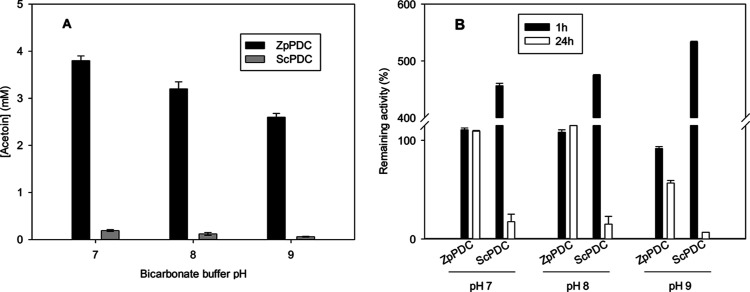
(A) Acetoin formation from acetaldehyde catalyzed by PDC at different pHs. Reaction conditions: bicarbonate buffer 250 mM, acetaldehyde 10 mM, TPP 1 mM, MgCl_2_ 1 mM, PDC 5 U mL^–1^ at 25 °C and 500 rpm agitation. Reaction time was 24 h. PDC from *Z. palmarum* (ZpPDC) and PDC from *S. cerevisiae* (ScPDC) were used independently. (B) Stability of PDC from *Z. palmae* (ZpPDC) and *S. cerevisiae* (ScPDC) during the reaction at different bicarbonate buffer pHs. Reaction conditions: bicarbonate buffer 250 mM, acetaldehyde 10 mM, TPP 1 mM, MgCl_2_ 1 mM, ZpPDC or ScPDC 5 U mL^–1^ at 25 °C, and 500 rpm agitation. Error bars correspond to standard deviation (*n* = 2).

When the synthesis of pyruvate was studied using PDC from *Z. palmae*, the desired ketoacid was not produced at any of the tested pHs. Otherwise, acetoin was detected in the sample analysis at all pH values, showing that PDC catalyzes an undesired side reaction that converts two molecules of acetaldehyde into acetoin, instead of forming pyruvic acid from CO_2_ and acetaldehyde (Figures S1 and S2). This side reaction has already been reported in the literature for other PDCs from *Zymomonas mobilis* and *Saccharomyces carlsbergensis*.^20−22^ According to previous research, the PDC enzyme catalyzes the decarboxylation of pyruvate through the TPP coenzyme. After pyruvate binds to TPP, CO_2_ is liberated and the formed TPP-bound acetaldehyde is susceptible to the addition of a second aldehyde, which results in the formation of acetoin. Moreover, as the results in this work also suggest, this acetoin synthesis can occur when either pyruvate or acetaldehyde are the substrates. On the other hand, the obtained results showed that PDC from *Z. palmae* catalyzes this secondary reaction with a higher reaction rate than the desired pyruvate synthesis (Figure 6A) to the extent that no pyruvic acid could be detected. For this reason, ScPDC was also tested in the synthesis of pyruvic acid aiming to find an enzyme with a higher specificity toward CO_2_. First, ScPDC was tested under the same reaction conditions. The results obtained show that PDC from *S. cerevisiae* produced approximately 20-fold less acetoin than PDC from *Z. palmae* (Figure 6A), indicating that using ScPDC can be more favorable for the synthesis of pyruvic acid. However, pyruvic acid was not detected under the tested conditions either. Regarding enzyme stability (Figure 6B), ScPDC showed an hyperactivation, increasing 5-fold the initial activity after 1 h of reaction, while PDC from *Z. palmae* is more stable at all pHs tested (Figure 6B).

### 3.3 PDC Coupled to LDH Reaction Using Gaseous CO_2_

**Figure 7:**
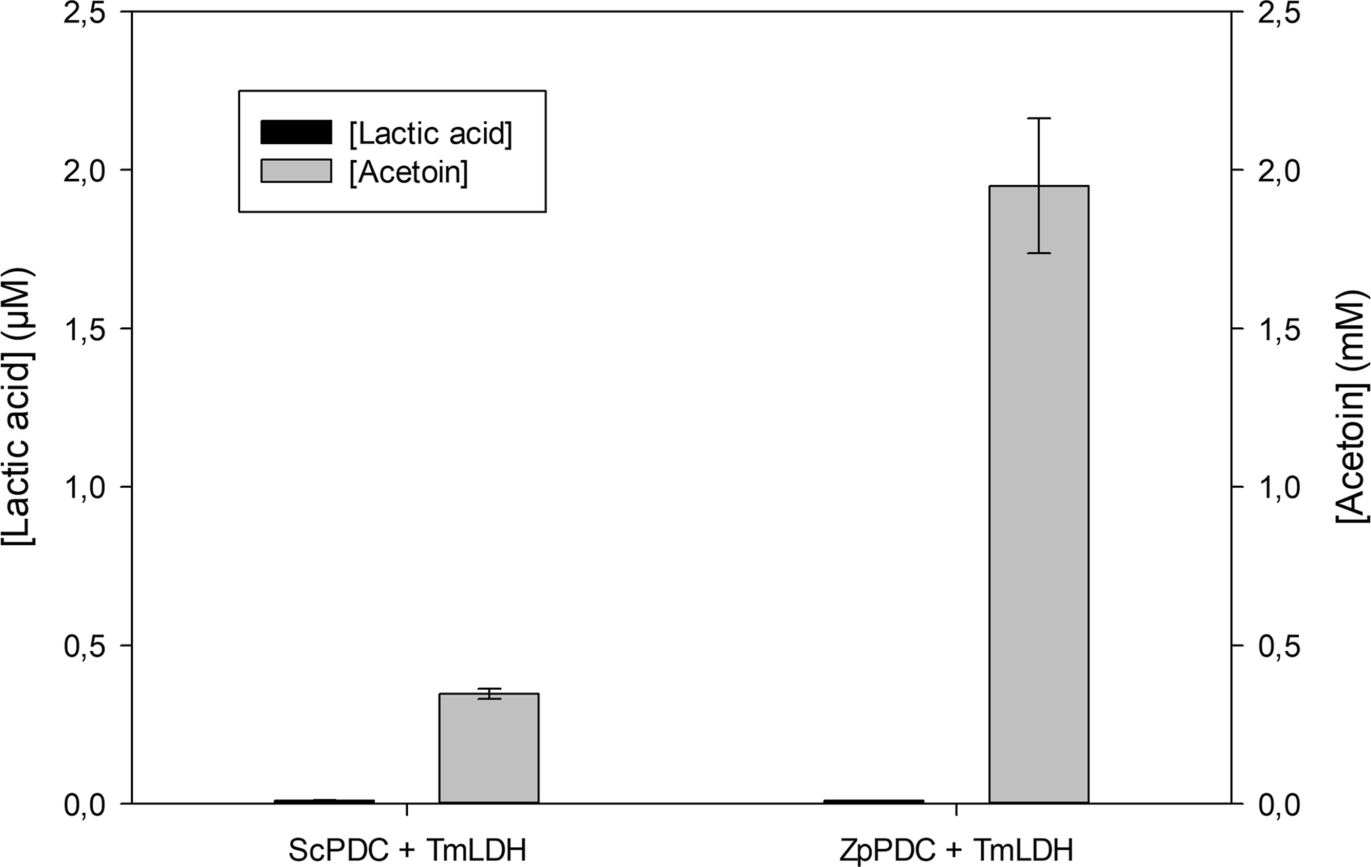
Lactate and acetoin concentration obtained with PDC variants coupled with LDH. Reaction conditions: phosphate buffer 250 mM at pH 7, acetaldehyde 5 mM, NADH 10 mM, TPP 1 mM, MgCl_2_ 1 mM, 1 atm of CO_2_, PDC 10 U mL^–1^, and LDH 10 U mL^–1^ at 25 °C and 500 rpm agitation. The reaction time was 24 h. PDC from *Z. palmae* (ZpPDC) and PDC from *S. cerevisiae* (ScPDC) were used independently. Error bars correspond to standard deviation (*n* = 2).

According to the results obtained in the PDC study, pyruvate could not be detected neither with PDC from *S. cerevisiae* nor with PDC from *Z. palmae*, meaning that a selection of the most suitable enzyme could not be performed. Therefore, the conditions studied for the reaction should be modified, aiming to shift the equilibrium toward the formation of the ketoacid. Thus, two strategies were considered: (i) introducing gaseous CO_2_ to reach 1 atm of 100% CO_2_ and (ii) coupling the LDH reaction, which is highly shifted toward lactic acid. Thus, lactic acid formation was analyzed in these experiments to evaluate the performance of both PDCs as suitable enzymes for the multienzymatic system. Moreover, the acetoin concentration was also analyzed as a parameter to consider in the enzyme selection. The reaction was performed at pH 7 to favor the synthesis of lactic acid by TmLDH (Figure 7). It should be mentioned that since CO_2_ gas is added to reach a 100% CO_2_ composition in the gas phase, phosphate buffer was used in these experiments instead of bicarbonate buffer to maintain the pH at 7.

The results show that ScPDC produces significantly less acetoin than ZpPDC under the same reaction conditions, showing that ZpPDC is more efficient for acetoin synthesis than ScPDC. However, no lactic acid was detected in any of the coupled reactions, even when using reaction conditions that should favor product formation (substrate increase and coupling of a reaction highly shifted toward product), suggesting how challenging it is to reverse the decarboxylation reaction. Finally, ScPDC was selected for further studies for the multienzymatic system development considering the lower amounts of byproduct obtained and the hyperactivation detected, which were promising features of this enzyme compared to the ZpPDC.

### 3.4 Complete Multienzymatic Reaction

#### 3.4.1 Enzyme Ratio Optimization

Once the ScPDC was selected as the final candidate, the complete multienzymatic reaction was tested at 1 atm of CO_2_. First, the enzyme ratio was studied since it has been reported as a key parameter on the overall performance of the multienzymatic systems.^23^

Since no lactic acid was detected when ScPDC was coupled to TmLDH, the initial activity of ScPDC was increased from 10 to 150 U mL^–1^ aiming to increase the reaction rate of the limiting step: the conversion of acetaldehyde to pyruvate. Then, ScADH/ScPDC and TmLDH/ScPDC ratios of 0.5, 1, and 1.25 were tested maintaining constant the initial ScPDC activity.

The obtained results are listed in Table 1. There is a strong effect of enzyme ratios on the lactic acid concentration. The lowest value (10.87 ± 1.19 μM) is obtained when both ScADH and TmLDH activities are higher than ScPDC (1 ScADH/ScPDC and 1 TmLDH/ScPDC). Similar results (15.54 ± 0.83 μM) are reached when ScADH/ScPDC was 1.25 and TmLDH/ScPDC was 0.5. On the other hand, the best results are obtained when ScADH activity is lower than ScPDC (0.5 ScADH/ScPDC) and TmLDH activity is higher than ScPDC, reaching 29.29 ± 0.30 μM of lactic acid.

**Table 1:** Lactic Acid Formation from Ethanol and CO_2_ Catalyzed by the Multi-Enzymatic System Formed by ScADH, ScPDC, and TmLDH at Different Enzyme Ratios of ScADH/ScPDC and TmLDH/ScPDC^a^

ScADH/ScPDC	TmLDH/ScPDC	lactic acid (μM)
1.25	0.50	15.54 ± 0.83
0.50	1.25	29.29 ± 0.30
1.00	1.00	10.87 ± 1.19

a Reaction conditions: phosphate buffer 250 mM pH 7, 1 atm CO_2_, ethanol 1 M, NAD^+^ 10 mM, MgCl_2_ 1 mM, TPP 1 mM at 25 °C and 500 rpm agitation, and 150 U mL^–1^ PDC. The reaction time was 24 h. ADH from *S. cerevisiae*, PDC from *S. cerevisiae*, and LDH from *T. maritima*.

These results suggest that two events should be promoted at the same time to increase lactic acid synthesis: PDC reaction rate should be favored over the formation of acetaldehyde, while a lactate synthesis rate should be high enough to shift the multienzymatic system toward product formation.

#### 3.4.2 Reaction Media Optimization

Reaction media engineering was applied to increase the process metrics. In that sense, other aqueous buffers were tested: Tris–HCl, citrate, and MOPS using the enzyme ratios and amounts that gave the highest lactic acid concentration in phosphate buffer, 0.5 ADH/PDC, 1.25 PDC/LDH, and 150 U mL^–1^ ScPDC.

**Figure 8:**
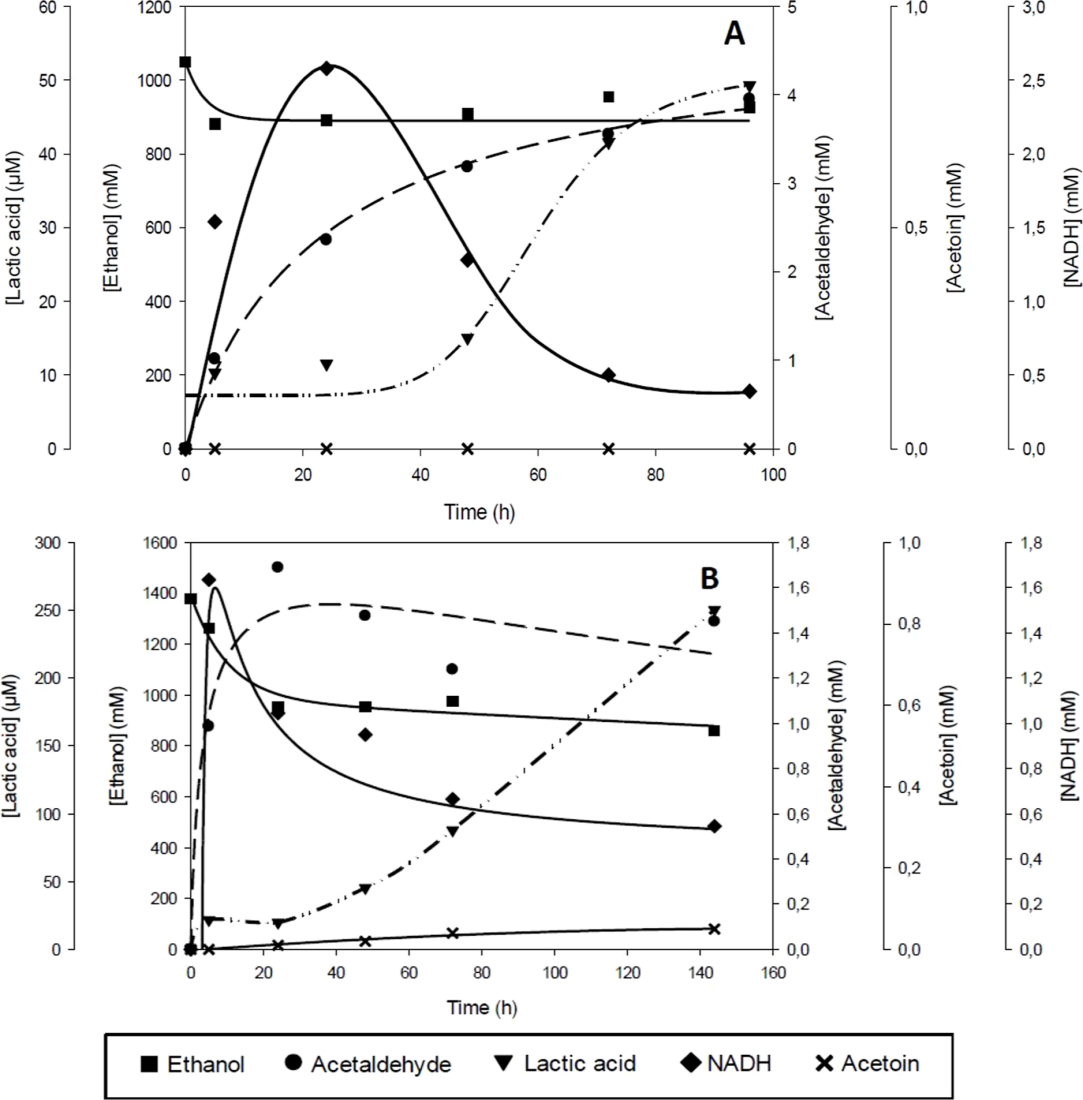
Concentration profile of substrate and products of the multienzymatic system over time. (A) Reaction performed using citrate buffer. Reaction conditions: citrate buffer 250 mM at pH 7, NAD^+^ 10 mM, ethanol 1 M, TPP 1 mM, MgCl_2_ 1 mM, 1 atm of CO_2_, ADH 75 U mL^–1^, PDC 150 U mL^–1^, and LDH 187.5 U/mL at 25 °C and 500 rpm agitation. The reaction time was 96 h. (B) Reaction performed using MOPS buffer. Reaction conditions: MOPS buffer 250 mM at pH 7, ethanol 1 M, TPP 1 mM, MgCl_2_ 1 mM, 1 atm of CO_2_, ADH 75 U mL^–1^, PDC 150 U mL^–1^, and LDH 187.5 U mL^–1^ at 25 °C and 500 rpm agitation. The reaction time was 144 h. ADH from *S. cerevisiae*, PDC from *S. cerevisiae* (ScPDC), and LDH from *T. maritima* were used.

The multienzymatic system performed with Tris–HCl led to no lactic acid formation. Thus, this buffer was discarded for further studies. Results using citrate buffer and MOPS are depicted in Figure 8A,B. Regarding the citrate buffer (Figure 8A), the lactic acid concentration follows a sigmoidal shape, which may be explained by the increase of acetaldehyde from 2.36 mM at 24 h to 3.95 mM at 96 h, where lactic acid has a second increase due to a raise of substrate (acetaldehyde) available for ScPDC. At the end of the reaction, lactic acid concentration reaches 49.24 μM, 1.7-fold higher compared to the result obtained with phosphate buffer. NADH has a peak concentration at 24 h (2.58 mM) and then decreases until the end of reaction, indicating that this cofactor is consumed at a higher rate than it is produced. However, the consumed NADH does not correspond to the lactic acid produced, therefore this decrease may correspond to NADH instability under the reaction conditions. It should be mentioned that ethanol decreases from 1050 to 900 mM at the very beginning of the reaction probably due to evaporation during CO_2_ sparging.

On the other hand, when the reaction was performed using MOPS buffer (Figure 8B), compared with citrate buffer, a similar profile is obtained. However, a higher lactic acid concentration was detected, reaching 250 μM at the end of the reaction, surpassing by 5-fold and 8.5-fold the concentration using citrate and phosphate, respectively (Figures S3 and S4). Taking into account that MOPS has a p*K*_a_ of 7.2,^24^ the reaction can be buffered at pH 7, with a final reaction pH of 6.6. As seen in previous experiments (Figures 2 and 3), ScPDC and TmLDH are most active and stable at pH 6–7. Since the reaction pH with MOPS is maintained in this range, ScPDC and TmLDH have a higher activity and stability, which could explain the higher production of lactic acid.

Higher concentrations of lactic acid were obtained using citrate buffer and MOPS compared to the results using phosphate buffer may be due to the ScPDC performance since it has been previously described that it is competitively inhibited by phosphates, with a relatively low *K*_ip_ (inhibition constant) of 14.7 mM.^25^

In previous works where this multienzymatic system was first described, a lactic acid concentration of 87 μM is reported as the maximum reached value.^26^ Therefore, the present work represents a step-forward on the multienzymatic systems for CO_2_ valorization into lactic acid thanks to the followed approach, leading to the highest lactic acid concentration ever reported up to date.

#### 3.4.3 Reaction Using Synthetic Gas Mixture Mimicking Real Iron and Steel Industry Off-Gases

In iron and steel production, about 248.4 Mtonnes of CO_2_ are being emitted each year.^29,30^ Steel works are optimized to achieve a tangible environmental improvement and a commercial benefit through the following: (i) increasing the share of electric arc furnaces and (ii) integrating the utilization of the process gases for energy generation. Though, it is an industrial activity that still represented approximately 6.7% of total world CO_2_ emissions.^31^

In order to test the feasibility of the multienzymatic system to be applied with real industrial off-gases, a synthetic gas mixture mimicking the blast furnace off-gas composition of iron and steel industry was tested (24.5% CO_2_, 46.6% N_2_, 23.9% CO, 1.2% O_2_, and 3.8% H_2_) (data provided by Arcelor Mittal in the frame of BIOCON-CO2 project). The conditions were those obtained previously after the reaction optimization.

**Figure 9:**
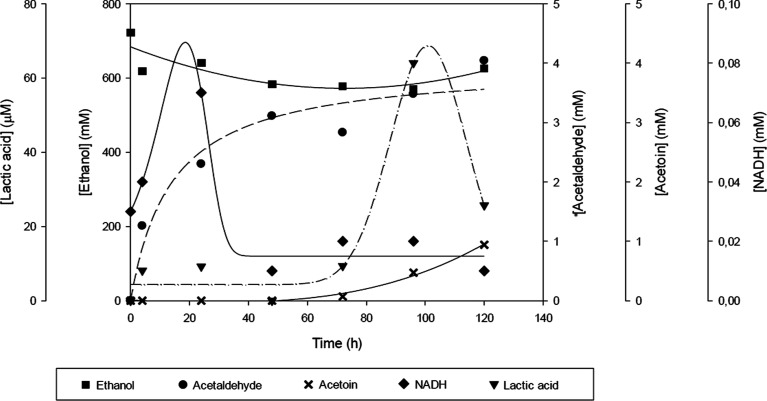
Concentration profile of the substrate and products of the multienzymatic system over time. Reaction conditions: MOPS buffer 250 mM at pH 7, ethanol 1 M, NAD^+^ 10 mM, TPP 1 mM, MgCl_2_ 1 mM, 1 atm of synthetic gas mixture, ADH 75 U mL^–1^, PDC 150 U mL^–1^, and LDH 187.5 U/mL at 25 °C and 500 rpm agitation. Reaction time was 120 h. ADH from *S. cerevisiae*, PDC from *S. cerevisiae* (ScPDC), and LDH from *T. maritima* were used.

As shown in Figure 9, the reaction may be divided into phases. In the first phase, ScADH enzyme consumes ethanol and NAD^+^ to produce acetaldehyde and NADH, which accumulates during the first hours of the reaction (up to 72 h). In the second phase, ScPDC and TmLDH begin to consume acetaldehyde to produce the final product, lactic acid, which reaches a concentration of 62 μM. It can be observed that during this second phase, the byproduct produced by the PDC begins to accumulate. This lower amount of lactic acid and the production of acetoin can be caused by a lower concentration of CO_2_ in the media. After one molecule of acetaldehyde binds to the TPP cofactor inside the active center of PDC, CO_2_ and a second molecule of acetaldehyde compete to produce pyruvic acid and acetoin, respectively. Therefore, this multienzymatic system shows that higher amounts of CO_2_ are required to favor the carboxylation reaction over the carboligation reaction. Moreover, NADH concentration decreases before lactic acid is produced, which unfavorably affects the last enzyme of the multienzymatic system, that requires NADH to reduce pyruvic acid into lactic acid. Despite these unfavorable conditions mimicking industrial gas composition, the system is still able to produce lactic acid.

## Supporting Information Available

Materials and methods and analysis of standards and reaction samples (PDF)
