# Supplementary material for: Lactic Acid from CO2: A Carbon Capture and Utilization Strategy Based on a Biocatalytic Approach
Source: Environ Sci Technol. 2023 Dec 11;57(51):21727–35. doi: 10.1021/acs.est.3c05455 (PMC10753888; doi:10.1021/acs.est.3c05455)
Supplement: Supplementary file 1 — es3c05455_si_001.pdf [file es3c05455_si_001.pdf]

Lactic acid from CO_2: a Carbon Capture and Utilization strategy based on a biocatalytic approach

Albert Carceller; Marina Guillén* and Gregorio Álvaro

Number of pages: 8

Figures: 4

Supporting information

Additional experimental details, materials, and methods

Enzyme production and purification

PDC from *Zymobacter palmae* (ZpPDC) was produced according to Alcover-Fortuny et al. (Alcover et al., 2019).

S. cerevisiae PDC (ScPDC), *S. cerevisiae* ADH (ScADH) and *T. maritima* LDH (TmLDH) were expressed in recombinant *Escherichia coli* M15 *ΔglyA* strains transformed with pVEF-HisScADH, pVEF-HisScPDC or pVEF-HisTmLDH, all enzymes were expressed with a His-tag. Bacterial cultures were performed as described by Benito, M. *et al.* (Benito et al., 2022). Briefly, a pre-inoculum culture was prepared by adding 100 µL of glycerol stocks to 20 mL of defined media (composition described by (Vidal et al., 2008)), and incubated overnight at 37°C with 150 rpm agitation. Inoculum cultures were prepared twice with 200 mL of defined media, inoculated with pre-inoculum at an initial OD600 of 0.2 and incubated at 37°C with 150 rpm agitation. When inoculum cultures reached an OD_{600} of 1.2, they were transferred to 2.5L of defined media prepared in a 5 L bioreactor to reach an initial OD_{600} of 0.2. An initial batch-phase was performed at 37°C, pH 7.0 and pO_2 30%. The pH was controlled with a NH_4OH 15% v v^{-1} solution. When glucose was consumed, the fed-batch phase was initiated with a preprogrammed exponential addition of feeding medium (composition described by (Vidal et al., 2008)), following the equation 1.

$$F = \frac{\mu \cdot X_O \cdot V_O \cdot e^{\mu \cdot \Delta t}}{Y_{X/S} \cdot S_O} \quad (1)$$

Where, F is feeding flow (L h^{-1}), X_0 is dry cell weight (g L^{-1}), V_0 is the working volume (L), μ is specific growth rate (h^{-1}), $Y_{X/S}$ is the biomass/substrate yield (g g^{-1}) and S_0 (carbon source concentration).

The induction phase was started when the culture reached an OD600 between 100 – 120, by the addition of IPTG at a 0.25 mM final concentration. Previously, temperature was decreased to 30°C to favor correctly folded recombinant protein. Induction phase ended when glucose accumulation was detected.

On the one hand, *Escherichia coli* cells expressing his-tagged enzymes were suspended in 50 mM phosphate buffer pH 7.5, 500 mM NaCl and 50 mM imidazole (supplemented with 0.5 mM TPP and 0.5 mM $MgCl_2$ for ScPDC enzyme). On the other hand, *E. coli* cells expressing ZpPDC were suspended in 50 mM acetate buffer pH 5 (supplemented with 0.5 mM TPP and 0.5 mM $MgCl_2$) Cell suspensions were disrupted using a cell disruptor (Constant Systems Ltd), followed by a centrifugation of the resulting homogenate (10,000g for 30 min at 4°C). Cell extracts were purified using an ÄKTA Pure 150 FPLC system (GE Healthcare®). As a first step, an affinity chromatography for His-tagged proteins, or anion-exchange chromatography for the non-His-Tagged ZpPDC was used. Then, the eluted proteins were buffer exchanged using a desalting resin. The affinity chromatography consisted in a Ni^{2+}-IDA-agarose equilibrated with a phosphate buffer 50 mM at pH 7.5, NaCl 500 mM and imidazole 50 mM (supplemented with 0.5 mM TPP and 0.5 mM $MgCl_2$ for PDC enzymes). The sample was injected followed with a column wash using the same equilibration buffer. The retained protein was eluted with a phosphate buffer 50 mM at pH 7.5, NaCl 500 mM and imidazole 500 mM (supplemented with 0.5 mM TPP and 0.5 mM $MgCl_2$ for PDC enzyme). The anion exchange chromatography consisted in a MANA-agarose equilibrated with an acetate buffer 50 mM

at pH 5 supplemented with 0.5 mM TPP and 0.5 mM $MgCl_2$. The sample was injected followed with a column wash using the same equilibration buffer. The retained protein was eluted with an acetate buffer 50 mM at pH 5 and 1M NaCl.

The eluted proteins from affinity chromatography and ZpPDC from anion exchange chromatography, were then injected after elution into a desalting column equilibrated with phosphate buffer 10 mM (supplemented with 0.5 mM TPP and 0.5 mM $MgCl_2$ for PDC enzymes) at pH 7.5. Finally, the purified proteins were stored overnight at -80ºC and lyophilized for 24h using a VirTis Freeze Drying equipment. Lyophilized protein powder was stored at -20ºC for long-term storage. For all the enzymes purified, all the fractions were loaded to NuPage 12% BisTris (Thermofisher Scientific®) following manufacturer instructions to assess purity and molecular weight. Protein concentration was measured by Bradford's method (Bradford, 1976), using Bovine serum albumin (BSA) as a standard.

References

Alcover, N., Carceller, A., Álvaro, G., Guillén, M., 2019. Zymobacter palmae pyruvate decarboxylase production process development: Cloning in Escherichia coli, fed-batch culture and purification. Eng. Life Sci. 19, 502–512. https://doi.org/10.1002/elsc.201900010

Benito, M., Román, R., Ortiz, G., Casablancas, A., Álvaro, G., Caminal, G., González, G., Guillén, M., 2022. Cloning , expression , and one - step purification / immobilization of two carbohydrate - binding module - tagged alcohol dehydrogenases. J. Biol. Eng. 8, 1–14. https://doi.org/10.1186/s13036-022-00295-8

Bradford, M.M., 1976. A rapid and sensitive method for the quantitation of microgram quantities of protein utilizing the principle of protein-dye binding. Anal. Biochem. https://doi.org/10.1016/0003-2697(76)90527-3

Vidal, L., López-Santín, J., Caminal, G., Ferrer, P., 2008. Development of an antibiotic-free plasmid selection system based on glycine auxotrophy for recombinant protein overproduction in Escherichia coli. J. Biotechnol. 134, 127–136. https://doi.org/10.1007/s13213-014-0950-8

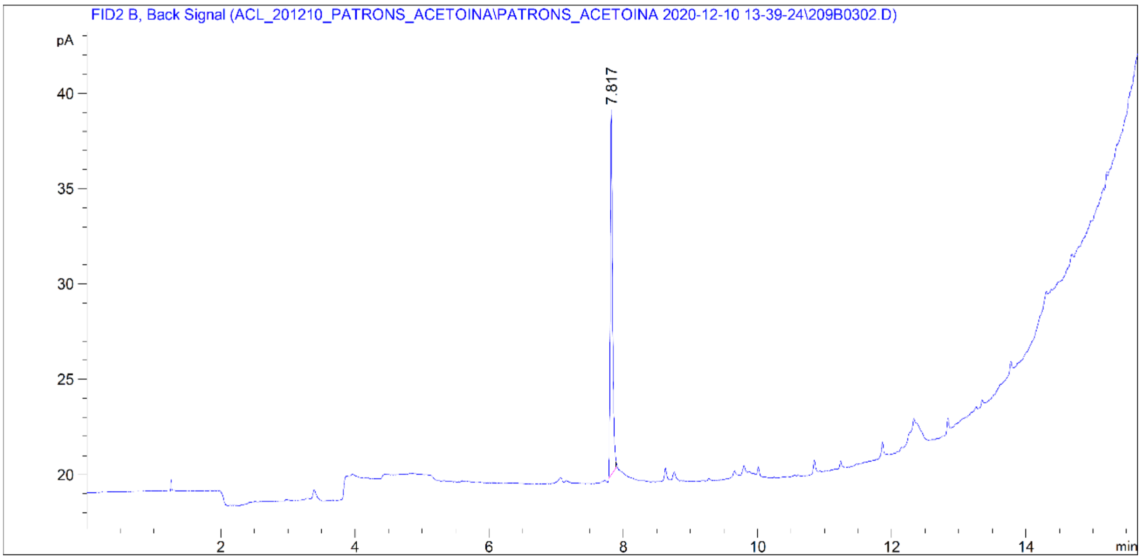

Figure S1. *Chromatogram of acetoin standard (0.7 mM). Analysis conditions: GC Shimadzu GC 2010 system, Stabilwax-DA column (15 m x 0.33 x 1 µm). Injection volume, 3 µL in split mode 20:1; injector temperature, 260°C; carrier gas, He at a constant flow of 3 mL min^{-1}. Initial oven temperature, 35°C, held for 2 min, then programmed to increase at 15°C min^{-1} to 120°C, and finally programmed to increase at 40°C min^{-1} to 240°C, held for 1 min. RT: 7. 8 min*

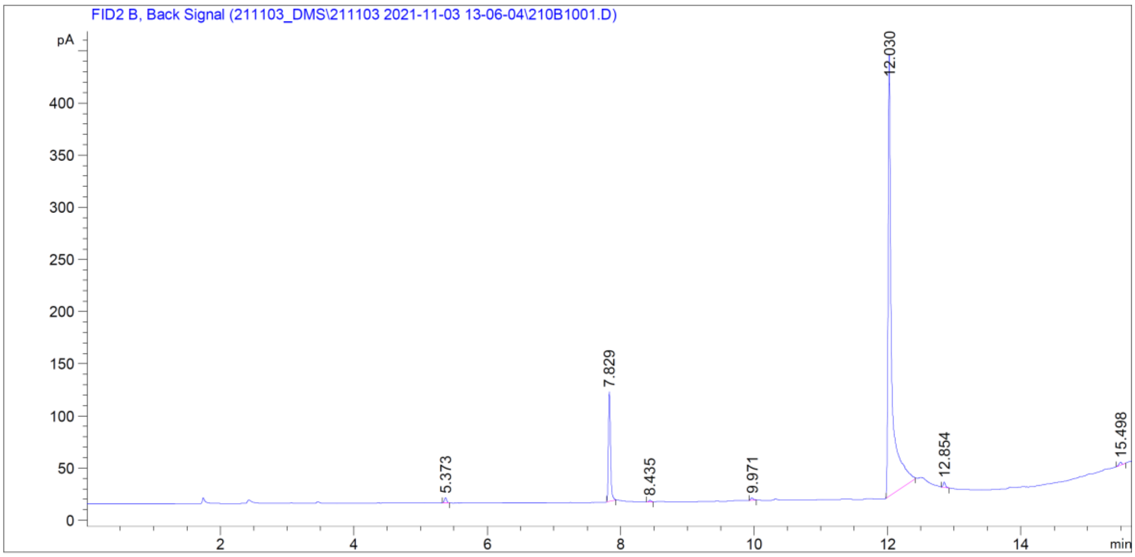

Figure S2. *Example of chromatogram of a PDC reaction sample containing acetoin (3.7 mM). Reaction conditions: bicarbonate buffer 250 mM at pH7, acetaldehyde 10 mM, TPP 1 mM, $MgCl_2$ 1 mM, ZpPDC 5 U mL^{-1} at 25°C and 500 rpm agitation. Reaction time 24 hours. Analysis conditions: Shimadzu GC 2010 system, Stabilwax-DA column (15 m x 0.33 x 1 µm). Injection volume, 3 µL in split mode 20:1; injector temperature, 260°C; carrier gas, He at a constant flow of 3 mL min^{-1}. Initial oven temperature, 35°C, held for 2 min, then programmed to increase at 15°C min^{-1} to 120°C, and finally programmed to increase at 40°C min^{-1} to 240°C, held for 1 min. Before the analysis reaction sample was inactivated adding 20 µL of 36% (v/v) HCl to 500 µL of sample. RT: 7. 8 min*

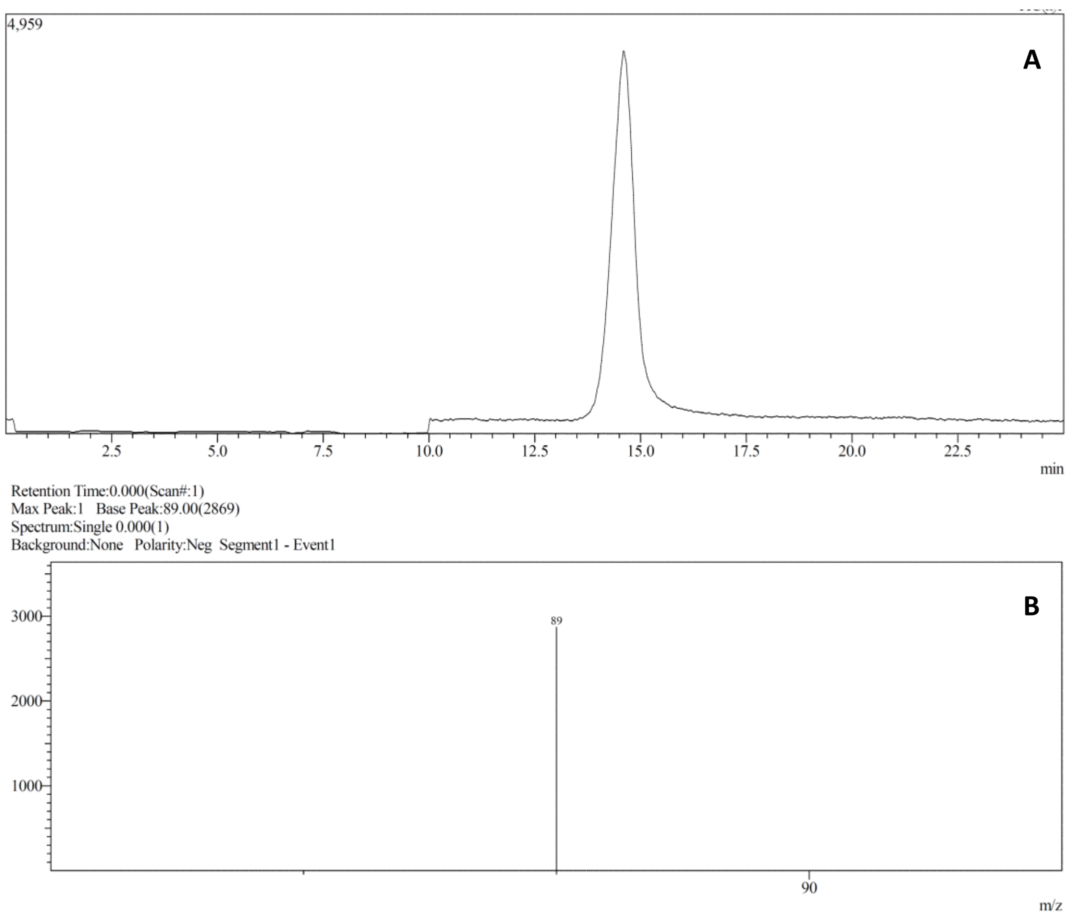

Figure S3. *Chromatogram (A) and spectrum (B) of a lactic acid standard (250 µM). Shimadzu LCMS-2010A using a ICSep 87H USP L17 (Transgenomic) column. The conditions were: buffer solution 640 µL L^{-1} of acetic acid, injection volume 5 µL, with a flow rate of 0.15 mL min^{-1}; the nebulizing gas was N_2 with a flow of 1.5L min^{-1}; the CDL temperature, 200°C; the heat block temperature, 200°C. The mass-to-charge ratio was set to 89 m/z to monitor the elution of lactic acid with a running time of 25 minutes. RT: 14. 8 min*

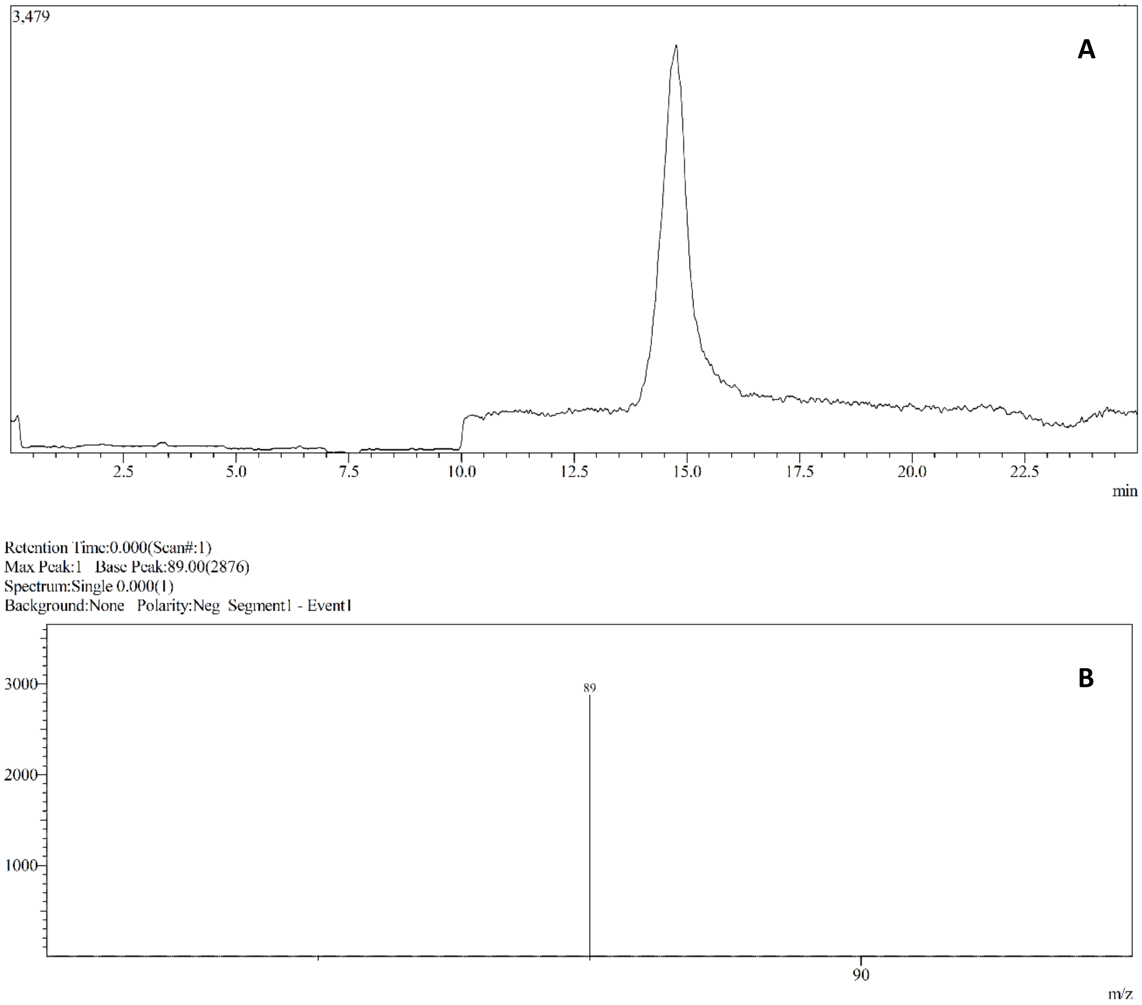

Figure S4. *Chromatogram (A) and spectrum (B) of a lactic acid (250 µM) obtained in the multi-enzymatic system using MOPS as buffer. Reaction conditions: MOPS buffer 250 mM at pH 7, ethanol 1M, TPP 1 mM, MgCl2 1 mM, 1 atm of CO2, ADH 75 U mL^{-1}, PDC 150 U mL^{-1}, LDH 187.5 U mL^{-1} at 25°C and 500 rpm agitation. Reaction time was 144 hours. ADH from S. cerevisiae, PDC from S. cerevisiae (ScPDC) and LDH from T. maritima were used. Analysis conditions: Shimadzu LCMS-2010A using a ICSep 87H USP L17 (Transgenomic) column. The conditions were: buffer solution 640 µL L^{-1} of acetic acid, injection volume 5 µL, with a flow rate of 0.15 mL min^{-1}; the nebulizing gas was N_2 with a flow of 1.5L min^{-1}; the CDL temperature, 200°C; the heat block temperature, 200°C. The mass-to-charge ratio was set to 89 m/z to monitor the elution of lactic acid with a running time of 25 minutes. RT: 14. 8 min*
